# Cold exposure and musculoskeletal conditions; A scoping review

**DOI:** 10.3389/fphys.2022.934163

**Published:** 2022-09-01

**Authors:** Erlend Hoftun Farbu, Anje Christina Höper, Eirik Reierth, Tohr Nilsson, Morten Skandfer

**Affiliations:** ^1^ Department of Community Medicine, UiT The Arctic University of Norway, Tromsø, Norway; ^2^ Department of Occupational and Environmental Medicine, University Hospital of North Norway, Tromsø, Norway; ^3^ Department of Library Services, UiT The Arctic University of Norway, Tromsø, Norway; ^4^ Department of Public Health and Clinical Medicine, Occupational and Environmental Medicine, Umeå University, Umeå, Sweden

**Keywords:** cold exposure, cold environment, cold temperature, musculoskeletal conditions, pain, regional pain, scoping review

## Abstract

**Background:** Musculoskeletal conditions are major contributors to years lived with disability. Cold exposure can be a risk factor, but any conclusion is obscure.

**Aim:** The aim of the present scoping review was to identify the existing evidence of an association between cold exposure and musculoskeletal conditions. The aim also included to consider pain in different regions and their assessment, as well as different measures of cold exposure, effect sizes, and to assess the feasibility of future systematic reviews and meta-analyses.

**Eligibility criteria:** The studies must have: an epidemiological design, defined cold exposure to come prior to the health outcome, defined exposure and outcome(s), existence of effect estimate(s) or data that made it possible to calculate such an estimate. Further, studies were required to be in English language and published in peer-reviewed journals. Studies that had a specific goal of studying cold exposure as an aggravator of already existing health problems were excluded.

**Sources:** We searched Ovid MEDLINE(R) and Epub Ahead of Print, In-Process and Other Non-Indexed Citations, Daily and Versions(R), and Embase Classic + Embase for original studies.

**Charting method:** The included studies were reviewed for study population, measurement of exposure and outcome, and effect size. Each publication was assessed for risk of bias.

**Results:** The included studies were heterogeneous in populations, measures of cold exposure and musculoskeletal conditions. Most studies used self-reported data. They were mostly cross-sectional studies, only two were prospective and one was a case-control study. Associations were found for different cold exposures and regional musculoskeletal conditions, but the heterogeneity and lack of studies impeded valid synthesis of risk magnitude, or meta-analyses.

**Conclusion:** The studies identified in this review indicate that cold exposure increases the risk of musculoskeletal conditions. However, there is a need for studies that better assess temporality between exposure and outcome. Future studies should also include better exposure assessment, including both objective measurements and measures of subjective experience of cold exposure. The heterogeneity in measurement of exposure and outcome impeded any meta-analysis.

## Introduction

Musculoskeletal conditions are among the most common causes of severe pain (Woolf and Pfleger, 2003) and are some of the leading contributors to the global burden of years lived with disability (Blyth et al., 2019). As a group, musculoskeletal conditions are estimated to cause 21.3% of the total years lived with disability in the world (Hoy et al., 2015). Most of the musculoskeletal conditions increase with age, and the present population age structure and the predicted increase in longevity imply augmented forthcoming occurrence. In addition, the risk of pain increases with age-related comorbidity, thus the global burden related to pain can be expected to continue to increase (Blyth et al., 2019).

Musculoskeletal conditions are highly diverse regarding aetiology, pathophysiology, anatomy, and impact on physical function. The collective concept entails all complaints related to muscles, joints, tendons, ligaments, and bone structures. The conditions may be systemic or regional. The latter include neuropathic disorders such as radiculopathies with pain or regional entrapment pain [e.g., Carpal tunnel syndrome (CTS)]. Many musculoskeletal conditions are defined by symptoms rather than clinical findings (e.g., low back pain), and pain is the major symptom. However, the aetiology of the pain can be difficult to identify (Treede et al., 2015). Consequently, the differentiation between musculoskeletal conditions and other pain conditions can be difficult. Therefore, pain or regional pain are often used terms.

The majority of studies on risk factors for musculoskeletal pain in the occupational context have explored the impact of job task, physical load, repetitiveness, static strain (NIOSH, 1997), or person related modifiers such as stress, anthropometry, BMI, vitamin status, or genetics (Mills et al., 2019). Concurrent exposures are the rule in epidemiological studies, obscuring any effects of the multitude of risk factors and modifiers not included in the study. The hypothesis claiming to account for the findings include metabolic mechanisms, pathologic muscle physiology, deteriorated blood supply or failing muscular control (Visser and van Dieën, 2006).

Original studies on cold environment as a risk factor for musculoskeletal conditions or pain are rare, and they are divergent in designs, assessment methods and case definitions. The human reaction to cold exposure is relative and relates to differences in temperature and is as such influenced by adaption, acclimatisation, coping and physical status. The impact relates to cold ambient temperature modified by wind chill, humidity, and contact cooling. Various studies use proxies for cold exposure either as measured temperature, estimated cooling effect, isotherms, climate, latitude, and contrasts from season, or outdoor/ indoor exposures.

The aim of the present scoping review was to identify the existing evidence of an association between cold exposure and musculoskeletal conditions. The aim also included to consider pain in different regions and their assessment, as well as different measures of cold exposure, effect sizes, and to assess the feasibility of future systematic reviews and meta-analyses.

## Materials and methods

This review was planned as a systematic review and registered in PROSPERO (ID: CRD42018108223). However, due to large heterogeneity in the measurement of exposure and outcome it became evident that it was not feasible to perform a systematic review. This will be described and discussed in Results and Discussion. It was therefore carried out as a scoping review. The following eligibility criteria were defined: epidemiological design, defined cold exposure to come prior to the health outcome, defined exposure and outcome(s), existence of effect estimate(s) or data that made it possible to calculate such an estimate. Further, studies were required to be in English language and published in peer-reviewed journals. Studies that had a specific goal of studying cold exposure as an aggravator of already existing health problems were excluded.

### Search

**FIGURE 1:**
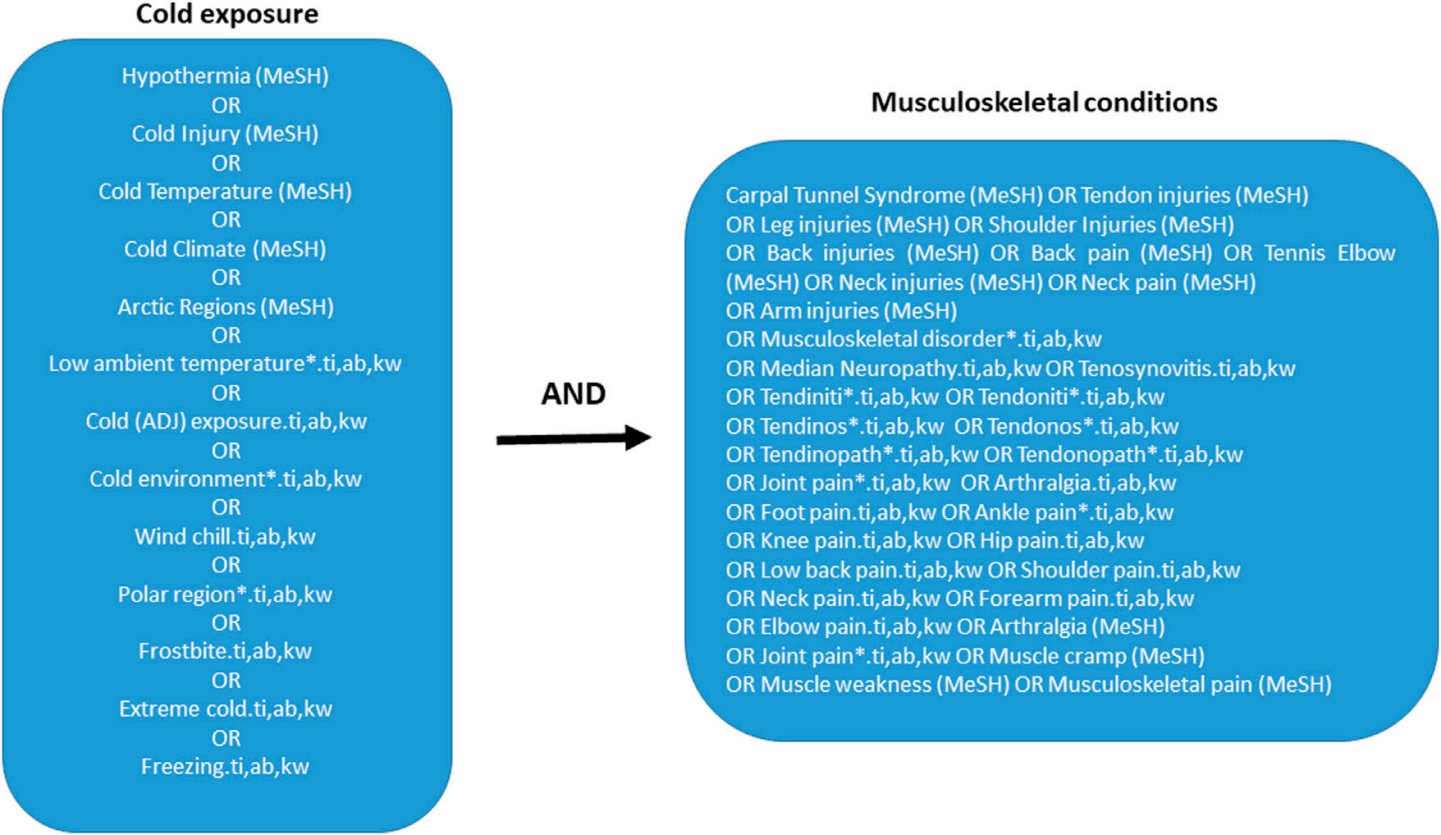
Systematic literature search of February 2022 in the following databases: Ovid MEDLINE(R) and Epub Ahead of Print, In-Process and Other Non-Indexed Citations, Daily and Versions(R), and Embase Classic + Embase. 1980 to Present. Medline subject headings; ti, titles; ab, abstracts; kw, keywords.

**FIGURE 2:**
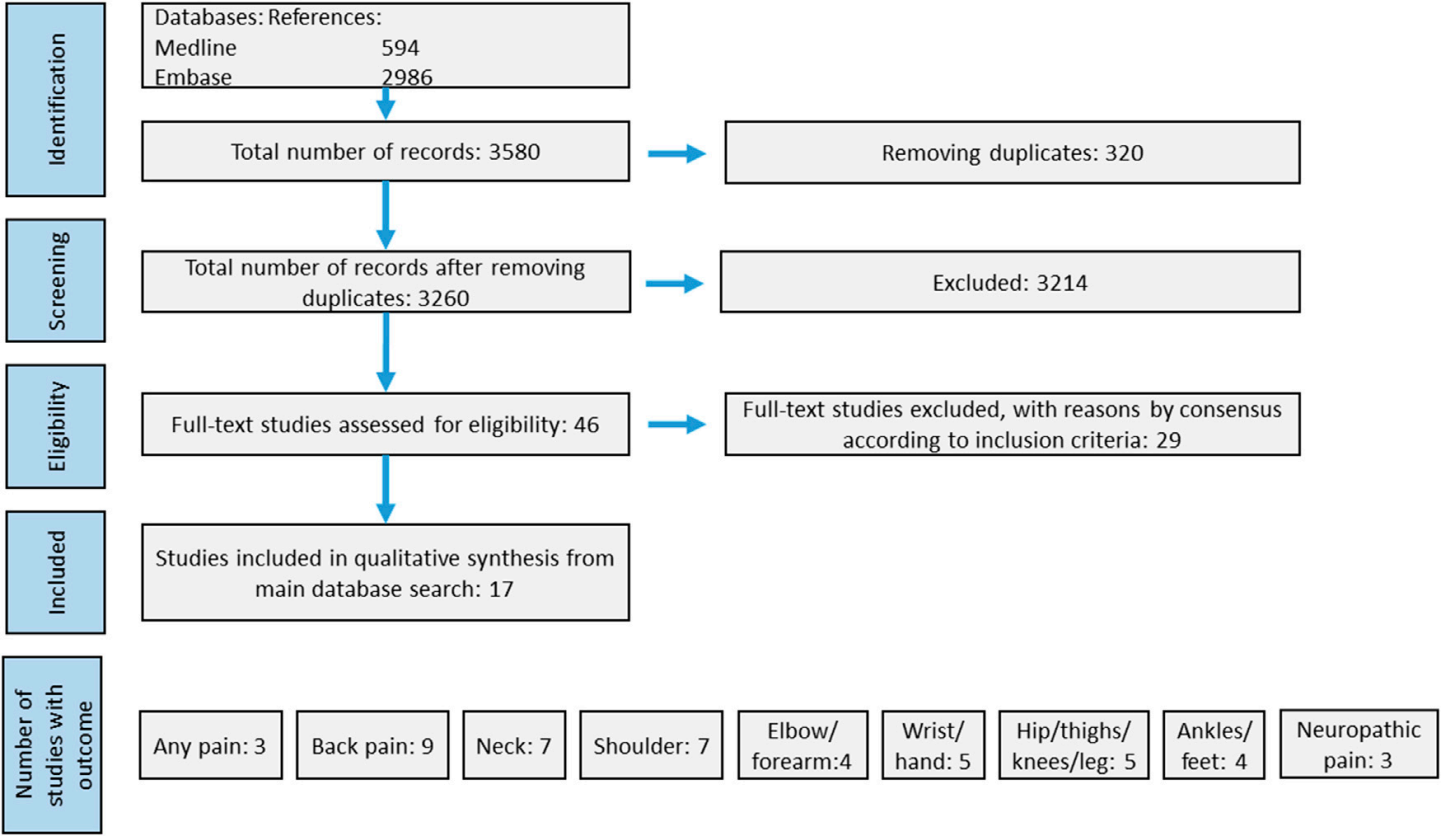
Prisma flow-chart displaying the literature search history. Of totally 3,580 references, we included 17 studies.

Cold exposure was defined by search terms addressing temperature, climate, region and cold effects, while health outcome was defined by concepts on musculoskeletal conditions subclassified by body regions. The search string developed in Medline and EMBASE is presented in Figure 1. Databases searched were Ovid MEDLINE(R) and Epub Ahead of Print, In-Process and Other Non-Indexed Citations, Daily and Versions(R), and Embase Classic + Embase. We used controlled vocabulary search terms (MeSH- and Emtree-index), whenever applicable. In addition, we used search fields, with title, abstract and keyword heading, as the preferred fields to search. Our search was performed with a time limit set for publications between the years 1980 and 2022. The search was last updated in February 2022. The identified publications were first assessed as titles and abstracts by reviewers ACH, MS, and TN for eligibility, and the approach from the preferred reporting items for scoping reviews statement was followed (Tricco et al., 2018). The number of studies at each stage of the process are shown in Figure 2. Based on the inclusion criteria, 46 studies were selected and considered in full text by four of the reviewers in a plenary session (ACH, MS, TN, and EHF). Any disagreement concerning eligibility for inclusion was resolved through joint discussion. Of the 46 titles, 17 were selected for inclusion (Figure 2). No studies were identified from other sources like reference lists of included studies.

### Data extraction

The 17 studies were examined by four authors (ACH, MS, TN, and EHF) in full text for obtaining type of epidemiological design, assessment of cold exposure and musculoskeletal condition. Further, the number of included subjects, incidence or prevalence, as well as association measures as odds ratio (OR), relative risk (RR) or incidence rate ratio (IRR) and confidence intervals (CI) were extracted by one author (EHF). Any missing effect estimate were calculated using the inbuilt risk-ratio/odds-ratio calculator in STATA 16. Exposure measures were classified either as using measured temperature or proxy for it, or self-reported cold exposure. The method of collecting outcomes was either by questionnaire or by a clinical examination. The outcomes were categorized according to regions. In addition, carpal tunnel syndrome (CTS) and lumbar radiculopathy were categorized as neuropathic pain.

### Critical appraisal of individual sources of evidence

To make it easier for the reader to judge the validity of the estimates provided we included an assessment of the included studies. The studies were assessed by the reviewers using a score system developed for this review (Supplementary Table S1). Sub-scores for method, exposure, and outcome were added up to a total score, a higher score meaning less risk of bias. Maximum points for the outcome sub-score were different for regional pain and neuropathic pain, leading to a potential maximum total score of 21 and 24, respectively (Supplementary Table S1). Any disagreements between the authors were resolved and concluded in joint sessions including four of the authors.

## Results

Out of 2,770 unique hits, 46 studies were assessed in full text (Figure 2). A total of 17 studies were identified as eligible for inclusion (Table 1). One of the studies provided data that contained what appears to be a logical flaw, there were more participants not working due to musculoskeletal pain than participants having musculoskeletal pain (Altuntas and Cankaya, 2020). We therefore chose to not calculate and present any effect estimates from this study.

**TABLE 1:** The included studies in alphabetical order.

Study	Population	Country	Design	Exposure	Comparison	Outcome
Altuntas and Cankaya (2020) ^a^	Poultry workers	Turkey	Cross-sectional	Number of years working in an environment <9°C	Less than 2 years	Self-reported, nordic questionnaire, neck, shoulder, elbow, wrist/hand, upper back, low back, hip/thighs, knees, ankles/feet
Bodin et al. (2012)	General working population	France	Cross-sectional	Self-experienced cold exposure >4 h/day	Self-experienced cold exposure ≤4 h/day	Rotator cuff syndrome diagnosed with clinical examination
Shoulder pain from Nordic Questionnaire						
Burstrom et al. (2013)	Male construction workers	Sweden	Cross-sectional	Geographical regions with different mean temperature	Warmest region	Self-reported back/neck pain last 12 months that have reduced work capacity
				North		
				Central		
Chiang et al. (1990)	Office workers, non-frozen food packers, frozen food packers	China	Cross-sectional	Handling frozen food (−12 to −15°C), or handling non-frozen food	Office workers	Carpal tunnel syndrome diagnosed with clinical and electrophysiological examination
Dovrat and Katz-Leurer (2007)	Store workers	Israel	Cross-sectional	Cold store work, −20°C	Store workers working in 20°–25°C	Self-reported, Nordic Questionnaire, low back pain
Farbu et al. (2019)	General working population	Norway	Cross-sectional	Self-reported working in cold environments ≥25% of the time	Self-reported working in cold environments <25% of the time	Self-reported chronic pain. Categorized into 1–2 pain sites, ≥3 pain sites, and at specific site neck, back, shoulder, arm, hand, hip, leg, foot
Farbu et al. (2021a)	General working population	Norway	Prospective	Self-reported working in cold environments ≥25% of the time	Self-reported working in cold environments <25% of the time	Musculoskeletal complaints (MSC) lasting 3 months or more
Ghani et al. (2020)	Store workers	Pakistan	Cross-sectional	Working in −20°C to −30°C	Working outdoors or in office	Musculoskeletal pain, adopted from the Nordic questionnaire
Milgrom et al. (2003)	Army recruits	Israel	Prospective	Winter	Summer	Clinically verified Achilles tendinosis or paratendinopathy
Piedrahí;ta et al. (2004)	Meat processing workers	Colombia	Cross-sectional	Ambient air temperature 2°C, measured at workplace	Ambient air temperature 11°C, measured at workplace	Self-reported, Nordic questionnaire
Pinar et al. (2013)	Ammunition factory workers	Turkey	Cross-sectional	Self-reported cold environment at workplace	No cold environment at workplace	Self-reported categorized as Yes/No
Pope et al. (1997)	General population	United Kingdom	Cross-sectional	Self-reported sometimes or always exposed to cold at work	Never exposed to cold at work	Self-reported, shoulder pain lasting 24 h or more last month
Raatikka et al. (2007)	General population	Finland	Cross-sectional	Self-reported cold exposure by hours/week exposed in past winter		Self-reported, repeated pain believed to be caused by cold
Skandfer et al. (2014)	Mine workers	Russia	Cross-sectional	Self-reported	Not working in <10°C ≥ 20 h a week	Self-reported low back pain, Nordic questionnaire
				Temperature at workplace <10°C ≥ 20 h a week	Not wet clothes >5 h a week	
				Wet clothes >5 h a week		
Sormunen et al. (2009)	Food processing workers	Finland	Cross-sectional	Perceiving slight/some/extensive cooling experience of body parts	Perceiving none cooling experience of body parts	Self-reported pain affecting activities of daily life
Stjernbrandt and Farbu (2022)	General working population	Sweden	Cross-sectional	Exposure to outdoor or cold environment at work reported on NRS from 0 (Do not agree) to 10 (Fully agree). Categorized into tertiles	First tertile	Self-reported neck pain, back pain, and lumbar radiculopathy
	General population	Sweden	Cross-sectional	Exposure to outdoor or cold environment in leisure time reported on NRS from 0 (Do not agree) to 10 (Fully agree). Categorized into tertiles	First tertile	Self-reported neck pain, back pain, and lumbar radiculopathy
Yagev et al. (2007)	Carpal tunnel syndrome patients scheduled for operation	Israel	Case-control	Self-reported cold environment at workplace	No cold environment at workplace	Carpal tunnel syndrome after clinical examination

a No effects sizes were calculated from this study due to a possible logical flaw in the provided data.

### Populations

Six of the studies recruited from the general population (Pope et al., 1997; Raatikka et al., 2007; Bodin et al., 2012; Farbu et al., 2019; Farbu et al., 2021a; Stjernbrandt and Farbu, 2022), one study recruited cases and controls from a clinic (Yagev et al., 2007), and the remaining 10 studies comprised of specific working populations, such as construction workers (Burstrom et al., 2013), miners (Skandfer et al., 2014), food processing workers (Piedrahí;ta et al., 2004; Sormunen et al., 2009; Altuntas and Cankaya, 2020), store workers (Chiang et al., 1990; Dovrat and Katz-Leurer, 2007; Ghani et al., 2020), army recruits (Milgrom et al., 2003) or factory workers (Pinar et al., 2013). The number of participants ranged from 162 to 118 258 (Tables 2, 3).

**TABLE 2:** Effect estimates sorted after type and type of assessment of neuropathic conditions.

	Outcome	Exposure		Effect estimate (95% CI)	Percent having outcome of total sample	Numbers of participants		Risk of bias	Study
						Exposed	Total		
Clinical examination	Carpal tunnel syndrome	Handling frozen food (−12 to −15°C)	Both genders	OR 9.39 (2.37–37–19)	30	121	207	16	Chiang et al. (1990)
		Self-reported cold environment at workplace	Both genders	OR 3.52 (1.08–11.47)	n.a	Not reported	229	15	Yagev et al. (2007)
Self-reported	Lumbar radiculopathy	Self-reported occupational exposure. Lowest tertile as reference medium (2. tertile) high (3. tertile)	Both genders	OR 1.29 (1.02–1.62)	6.2	2093	8,740	6	Stjernbrandt and Farbu (2022)
				OR 1.36 (1.07–1.73)		1958			
		Self-reported leisure-time exposure. Categorized into tertiles. Highest compared to lowest	Both genders	OR 1.15 (0.91–1.44)	Not reported	Not reported	12 627	6	Stjernbrandt and Farbu (2022)

OR: Odds Ratio CI: confidence interval.

**TABLE 3:** Effect estimates sorted after type and type of assessment of pain or regional pain.

Outcome		Exposure		Effect estimate (95% CI)	Percent having outcome of total sample	Numbers of participants		Risk of bias	Study
						Exposed	Total		
Clinical examination	Rotator cuff syndrome	Self-reported cold exposure >4 h/day	Men	Not reported	7.0	149	2,161	11	Bodin et al. (2012)
			Women	OR 1.3 (0.5–3.5)	9.3	71	1,548		
	Achilles paratendinitis	Winter compared to summer	Men	RR 2.62 (1.71–4.01)^a^	6.8	697	1,500	10	Milgrom et al. (2003)
Self-reported pain	Any MSC	Self-reported working in cold environments ≥25% of the time	Both genders	IRR 1.15 (1.03–1.29)	55.7	258	2,347	12	Farbu et al. (2021a)
	Severe MSC			IRR 0.95 (0.60–1.48)	8.4				
	MSC in ≥3 regions			IRR 1.11 (0.83–1.49)	17.6				
	Any pain	Self-reported work in cold environment	Both genders	OR 1.84 (1.37–2.47)	39.3	323	955	8	Pinar et al. (2013)
		Per 10 h/week cold exposure	Men	OR 1.13 (1.03–1.22)	30.2	n.a	2,332	8	Raatikka et al. (2007)
			Women	OR 1.35 (1.14–1.58)	27.2		2,840		
	Pain at 1-2 sites	Self-reported working in cold environments ≥25% of the time	Both genders	OR 0.95 (0.73–1.24)	16.3	623	5493	8	Farbu et al. (2019)
	Pain at ≥3 sites			OR 1.57 (1.23–2.01)	18.7	666	5657		
	Low back	Working in −20°C compared to >20°C	Men	OR 2.6 (1.03–6.5)	32.8	64	122	10	Dovrat and Katz-Leurer (2007)
		North central	Men	OR 1.19 (1.14–1.24)	24.3	23 514	118 258	10	Burstrom et al. (2013)
				OR 1.09 (1.05–1.13)		57 148			
		Wet clothes ≥5 h <10°C working environment	Men	OR 1.54 (1.31–1.81)	51	1,196	3,530	9	Skandfer et al. (2014)
				OR 1.81 (1.54–2.14)		1,668			
		Working in 2°C compared to 8–12°C	Men	PR 4.48 (1.61–12.4)	9.3	50	112	9	Piedrahí;ta et al. (2004)
		Experience of cooling at work	Both genders	OR 3.22 (2.28–4.55)	70	195	1,117	9	Sormunen et al. (2009)
		Slight		OR 6.08 (4.01–9.24)		175			
		Some		OR 7.36 (3.99–13.58)		78			
		Extensive							
		Per 10 h/week cold exposure	Men	OR 1.17 (1.04–1.30)	6.7	n.a	2,267	8	Raatikka et al. (2007)
			Women	OR 1.41 (1.04–1.87)	5.0		2,785		
		Self-reported working in cold environments ≥25% of the time	Both genders	OR 1.18 (0.91–1.52)	14.3	779	6,553	8	Farbu et al. (2019)
		Self-reported occupational exposure. Lowest tertile as reference	Both genders	OR 1.10 (0.94–1.29)	14.9	2093	8740	7	Stjernbrandt and Farbu (2022)
		Medium (2. tertile)		OR 1.38 (1.17–1.63)		1958			
		High (3. tertile)							
		Self-reported leisure-time exposure. Categorized into tertiles. Highest compared to lowest	Both genders	OR 1.01 (0.86–1.18)	Not reported	Not reported	12 627	7	Stjernbrandt and Farbu (2022)
		Cold stores workers (<−20°C) compared to normal	Men	RR 4.11 (2.66–6.34)	Not reported	100	200	6	Ghani et al. (2020)
	Upper back	Working in 2°C compared to 8–12°C	Men	PR 2.24 (0.32–15.45)	2.5	50	112	9	Piedrahí;ta et al. (2004)
		Cold stores workers (<−20°C) compared to normal	Men	RR 21.00 (6.82–64.65)	Not reported	100	200	6	Ghani et al. (2020)
	Neck	North	Men	OR 1.57 (1.47–1.67)	8.6	23 514	118 258	10	Burstrom et al. (2013)
		Central		OR 1.18 (1.12–1.25)		57 148			
		Working in 2°C compared to 8–12°C	Men	PR 11.2 (1.34–93.4)	3.7	50	112	9	Piedrahí;ta et al. (2004)
		Self-reported working in cold environments ≥25% of the time	Both genders	OR 1.46 (1.13–1.89)	13.7	779	6,553	8	Farbu et al. (2019)
		Self-reported occupational exposure. Lowest tertile as reference	Both genders	OR 1.15 (0.99–1.34)	17.5	2093	8,740		Stjernbrandt and Farbu (2022)
		Medium (2. tertile)		OR 1.36 (1.16–1.59)		1958			
		High (3. tertile)							
		Self-reported leisure-time exposure. Categorized into tertiles. Lowest as reference	Both genders	OR 1.10 (0.95–1.28)	Not reported	Not reported	12 627	7	Stjernbrandt and Farbu (2022)
		Cold stores workers (<−20°C) compared to normal	Men	RR 15.00 (6.33–35.51)	Not reported	100	200	6	Ghani et al. (2020)
	Neck/shoulder	Experience of cooling at work	Both genders	OR 2.28 (1.48–3.50)	84	202	1,117	9	Sormunen et al. (2009)
		Slight		OR 3.88 (2.49–6.05)		283			
		Some		OR 10.33 (4.81–22.19)		163			
		Extensive							
	Head/neck	Per 10 h/week cold exposure	Men	OR 1.12 (0.98–1.26)	10	n.a	2,293	8	Raatikka et al. (2007)
			Women	OR 1.09 (0.86–1.37)	10.8		2,821		
	Shoulder	Experience of cooling at work	Both genders	OR 2.77 (1.99–3.85)	65	177	1,117	9	Sormunen et al. (2009)
		Slight		OR 6.05 (4.03–9.10)		161			
		Some		OR 11.28 (4.69–27.15)		45			
		Extensive							
		Working in 2°C compared to 8–12°C	Men	PR 4.48 (0.85–23.6)	3.7	50	112	9	Piedrahí;ta et al. (2004)
		Per 10 h/week cold exposure	Men	OR 1.31 (1.12–1.51)	4.9	n.a	2,288	8	Raatikka et al. (2007)
			Women	OR 1.16 (0.81–1.98)	4.7		2,812		
		Self-reported cold exposure >4 h/day	Men	Not reported	28.0	149	2,161	8	Bodin et al. (2012)
			Women	OR 2.2 (1.3–3.8)	31.1	71	1,548		
		Occupational exposure	Men	RR 1.8 (0.6–5.4)	1	29	113	8	Pope et al. (1997)
		Occasional		RR 6.4 (1.5–27)	4.2	4			
		Always							
		Occasional	Women	RR 1.4 (0.4–5.1)	18.7	14	123		
		Always		RR 1.1 (0.2–8−5)		5			
		Self-reported working in cold environments ≥25% of the time	Both genders	OR 1.39 (1.08–1.78)	13.7	779	6,553	8	Farbu et al. (2019)
		Cold stores workers (<−20°C) compared to normal	Men	RR 151.00 (9.48–2,403.28)	Not reported	100	200	6	Ghani et al. (2020)
	Elbows	2°C compared to 8–12°C	Men	PR 2.24 (0.14–35.1)	1.2	50	112	9	Piedrahí;ta et al. (2004)
	Elbows/forearms	Per 10 h/week cold exposure	Men Women	OR 1.15 (0.94–1.45)	2.1	n.a	2,269	8	Raatikka et al. (2007)
				OR 1.30 (0.79–1.98)	2.1		2,800		
	Arm	Self-reported working in cold environments ≥25% of the time	Both genders	OR 1.34 (0.98–1.83)	8.4	779	6,553	8	Farbu et al. (2019)
	Elbows	Cold stores workers (<−20°C) compared to normal	Men	RR 10.40 (4.33–2,434.82)	Not reported	100	200	6	Ghani et al. (2020)
	Wrist	Experience of cooling at work	Both genders	OR 2.74 (1.78−4–22)	70	54	1,117	9	Sormunen et al. (2009)
		Slight		OR 6.11 (4.01–9.31)		72 90			
		Some		OR 20.12 (11.29–35.85)					
		Extensive							
	Wrists/hands	2°C compared to 8–12°C	Men	PR 2.24 (0.58–8.6)	4.9	50	112	9	Piedrahí;ta et al. (2004)
	Wrists/palm	Per 10 h/week cold exposure	Men Women	OR 1.11 (0.94–1.34)	5.6	n.a	2,268	8	Raatikka et al. (2007)
				OR 1.51 (1.18–1.91)	6.9		2,795		
	Hand	Self-reported working in cold environments ≥25% of the time	Both genders	OR 1.16 (0.79–1.71)	6	779	6,553	8	Farbu et al. (2019)
	Fingers	Per 10 h/week cold exposure	Men Women	OR 1.11 (0.95-1–27)	18.4	n.a	2,309	8	Raatikka et al. (2007)
				OR 1.34 (1.11–1.60)	16.6		2,815		
	Wrists/hands	Cold stores workers (<−20°C) compared to normal	Men	RR 23.33 (7.59–71.64)	Not reported	100	200	6	Ghani et al. (2020)
	Knees	2°C compared to 8–12°C	Men	PR 1.49 (0.26–8.66)	3.1	50	112	9	Piedrahí;ta et al. (2004)
	Hip	Self-reported working in cold environments ≥25% of the time	Both genders	OR 1.26 (0.90–1.75)	8.9	779	6,553	8	Farbu et al. (2019)
	Knees/thighs/calves	Per 10 h/week cold exposure	Men Women	OR 1.06 (0.90–1.23)	6.8	n.a	2,268	8	Raatikka et al. (2007)
				OR 1.13 (0.85–1.46)	7.6		2,793		
	Leg	Self-reported working in cold environments ≥25% of the time	Both genders	OR 1.47 (1.10–1.96)	10	779	6,553	8	Farbu et al. (2019)
	Hips/thighs	Cold stores workers (<−20°C) compared to normal	Men	RR 111.00 (6.95–1772.51)	Not reported	100	200	6	Ghani et al. (2020)
	Knees	Cold stores workers (<−20°C) compared to normal	Men	RR 6.87 (3.45–13.67)	Not reported	100	200	6	Ghani et al. (2020)
	Ankles/feet	2°C compared to 8–12°C	Men	PR 2.24 (0.14–35.1)	1.2	50	112	9	Piedrahí;ta et al. (2004)
		Per 10 h/week cold exposure	Men Women	OR 1.16 (1.03–1.30)	11	n.a	2,278	8	Raatikka et al. (2007)
				OR 1.34 (1.08–1.64)	11.6		2,808		
	Foot	Self-reported working in cold environments ≥25% of the time	Both genders	OR 0.8 (0.54–2.04)	6.6	779	6,553	8	Farbu et al. (2019)
	Ankles/feet	Cold stores workers (−20°C to −30°C) compared to normal	Men	RR 3.53 (2.13–5.83)	Not reported	100	200	6	Ghani et al. (2020)

PR, Prevalence ratio; RR, Relative risk; OR, Odds ratio; IRR, Incidence rate ratio; CI, Confidence interval aCalculated by the authors of this review.

### Measurement of exposure

There were two main categories of exposure measure: temperature and self-reported exposure to cold conditions. Seven studies used temperature as a measure of cold exposure. The measures were heterogenous, ranging from categories based on contact with frozen items (Chiang et al., 1990), to categories based on seasons (Milgrom et al., 2003), ambient air temperature measured at the workplace (Piedrahí;ta et al., 2004; Dovrat and Katz-Leurer, 2007; Ghani et al., 2020), self-reported number of years worked in an environment below 9°C (Altuntas and Cankaya, 2020), or different regions by latitude, implying different mean temperatures between the regions (Burstrom et al., 2013). Regarding level of exposure, one of the ten studies using self-reported exposure used weekly average time of cold exposure (Raatikka et al., 2007), while many self-reported cold exposures were specified with a certain minimum time of cold exposure per day, or week. For example, cold environment at the workplace >4 h pr day (Bodin et al., 2012), temperature at workplace <10°C ≥ 20 h a week or wearing wet clothes >5 h a week (Skandfer et al., 2014), working ≥25% of the time in cold environment (Farbu et al., 2019; Farbu et al., 2021a). One study used never, occasional or always exposed to cold environment at work (Pope et al., 1997), and two used self-reported cold environment at the workplace without assessing the duration or frequency of exposure (Yagev et al., 2007; Pinar et al., 2013). Another study assessed self-reported exposure to outdoor or cold environment and participants reported on a numerical rating scale from 0 (Do not agree) to 10 (Fully agree) for both occupational exposure and leisure time exposure. Only one study specified cold environment with a temperature threshold (Skandfer et al., 2014), while in one study participants were not asked about the environment, but the perceived cooling of different body parts (Sormunen et al., 2009). Most of the studies focused on occupational exposure. However, in one study participants were asked specifically about leisure time cold exposure as well as occupational exposure (Stjernbrandt and Farbu, 2022), and one used total time exposed to cold during a week (Raatikka et al., 2007). The studies using season or geographical regions also include differences in leisure time exposure (Milgrom et al., 2003; Burstrom et al., 2013).

### Assessments of outcome

Most of the studies used several different outcomes. The outcome measures could roughly be divided into neuropathic (Table 2) or musculoskeletal pain (Table 3). The neuropathic outcomes were clinically verified carpal tunnel syndrome in two studies (Chiang et al., 1990; Yagev et al., 2007), and self-reported lumbar radiculopathy in one (Stjernbrandt and Farbu, 2022). 15 of the studies included musculoskeletal pain as outcome. Two of these studies included a diagnosis verified by clinical examination; one used achilles paratendinitis and another rotator cuff syndrome (RCS) (Milgrom et al., 2003; Bodin et al., 2012). A total of 14 studies used self-reported musculoskeletal pain, either pain in general or at different sites. Six studies used questions from the Nordic Questionnaire to assess the 12-months prevalence of pain, either at multiple sites (Piedrahí;ta et al., 2004; Altuntas and Cankaya, 2020; Ghani et al., 2020), or at specific sites such as low back (Dovrat and Katz-Leurer, 2007; Skandfer et al., 2014) or shoulder (Bodin et al., 2012). In some of the studies participants were asked about pain that reduced work ability (Burstrom et al., 2013; Altuntas and Cankaya, 2020) or caused a disadvantage in daily activities (Sormunen et al., 2009). In one study participants were asked about shoulder pain lasting more than 24 h during the last month (Pope et al., 1997), and two studies specified that the pain should have lasted 3 months or more (Farbu et al., 2019; Farbu et al., 2021a), thereby more specifically assessing chronic pain. In one study participants were asked to report repeated musculoskeletal pain believed to be caused by cold temperature (Raatikka et al., 2007).

### Critical appraisal of individual sources of evidence

The risk of bias in the included studies was assessed by a scoring system developed for this review (Supplementary Table S1). The total score is presented in Tables 2, 3. The sub scores on exposure, outcome and method are presented in Supplementary Table S2. Most of the studies had less than half of the possible maximum scores, indicating a higher risk of bias. Fourteen of the studies had cross-sectional design, two were cohort studies (Milgrom et al., 2003; Farbu et al., 2021a) and one was a case-control study (Yagev et al., 2007).

### Results in the included studies

There were several different outcomes in many of the studies, and consequently a total of 85 effect estimates were extracted from the included studies (Tables 2, 3). Most of the estimates in the included studies showed an association between cold exposure and pain, either neuropathic or musculoskeletal pain (Tables 2, 3). However, not all were statistically significant, many of the estimates had wide confidence intervals and the estimates between studies varied.

The two studies on CTS found increased odds for cold exposure, either measured as self-reported cold environment at work (Yagev et al., 2007), or as handling frozen food (Chiang et al., 1990). In one study, the highest tertile of occupational cold exposure had significantly increased odds for lumbar radiculopathy, but not the highest tertile of leisure time cold exposure (Table 2) (Stjernbrandt and Farbu, 2022).

Among the studies using pain in general as an outcome, a prospective cohort study found a significantly increased risk of having any musculoskeletal complaints after 7–8 years (Farbu et al., 2021a). However, there were no significantly increased risks of severe or widespread musculoskeletal complaints. This contrasts the earlier findings from a cross-sectional analysis from the same authors showing a significant association between cold exposure and pain at ≥3 sites, but not for pain at 1–2 sites. A significant association between cold exposure and general musculoskeletal pain was also found in a sample of workers from a Turkish ammunition factory (Pinar et al., 2013), as well as between cold exposure and pain believed to be caused by cold exposure in a general population in Finland (Raatikka et al., 2007).

Most studies found cold exposure to be significantly associated with back pain (Table 3). The associations were found using self-reported exposure, subjective experience, geographical regions, and measured temperature at the workplace (Table 3). The results for upper back (including 4 cases) (Piedrahí;ta et al., 2004) and back pain lasting ≥3 months (Farbu et al., 2019) were not statistically significant. One study did not find any association between leisure time cold exposure and back pain (Stjernbrandt and Farbu, 2022)

Six of the seven studies using neck pain as an outcome found statistically significant associations between cold exposure and neck pain. The associations were found using self-reported exposure, subjective experience, geographical regions and measured temperature at the workplace (Table 3). Three of the studies consisted of reasonable large sample sizes from 6,533 to 118 258. Even though one study found a statistically significant association for occupational cold exposure, the association for leisure time cold exposure was not significant (Stjernbrandt and Farbu, 2022). Another study that did not find a statistically significant association had asked participants for head/neck pain the participants believed to be caused by cold exposure (Raatikka et al., 2007).

A total of seven studies included shoulder pain as outcome. One study of French employees showed that being exposed to cold >4 h per day was positively associated with self-reported shoulder pain in women, but not with clinically diagnosed RCS. The results for men were not reported because the authors did not find a statistically significant higher prevalence of shoulder pain with or without RCS among the 149 men exposed to cold environment (Bodin et al., 2012). Two other studies found a significant association for men, but not for women (Pope et al., 1997; Raatikka et al., 2007). One study found that those working in cold environment ≥25% of the time had significantly increased odds for shoulder pain lasting ≥3 months (Farbu et al., 2019).

Four studies included pain in elbows, forearms, or arms as outcomes (Piedrahí;ta et al., 2004; Raatikka et al., 2007; Farbu et al., 2019; Ghani et al., 2020). Even though all four studies found increased odds for cold exposure, only one was statistically significant. Studies of pain in the wrist, hands or fingers also consistently found increased odds for pain for cold exposure, but not all associations were statistically significant (Table 3). One study found significantly higher odds for pain in wrist or fingers believed to be caused by cold with increasing cold exposure among women, but not among men (Raatikka et al., 2007).

Among the five studies using hip, knees, thighs, calves or leg, two studies found associations: one between working in cold environments ≥25% of the time and pain in the leg lasting ≥3 months or more (Farbu et al., 2019), and one found significant associations between cold store work and pain in the hip/thighs and knees (Ghani et al., 2020).

There were four studies using ankle and/or foot as outcomes (Piedrahí;ta et al., 2004; Raatikka et al., 2007; Farbu et al., 2019; Ghani et al., 2020). One of the studies with the highest score in the critical appraisal found a higher incidence of achilles paratendinitis among military recruits trained in winter compared to summer (Milgrom et al., 2003), and one study reported an association between hours exposed to cold environment and pain in ankles or feet believed to be caused by cold exposure (Raatikka et al., 2007). One study found no association between cold exposure and pain in the foot lasting ≥3 months (Farbu et al., 2019).

## Discussion

Taken together, the limited evidence available indicates that cold exposure increases the risk of musculoskeletal conditions. The studies on cold exposure as a risk factor for CTS had the least risk of bias and found strong associations (OR of 3.52 and 9.39), but they were limited in size. Associations between cold exposure and back, neck and shoulder pain were found in several studies with reasonable large sample sizes. However, the studies included in this review are in general at risk of being biased. The majority was cross-sectional and only two studies were prospective, in addition to one case-control study. One of the prospective studies attained the outcome 7–8 years after the measurement of exposure and did not include information about the status of exposure at follow-up. Further, most included studies use self-reported exposures or outcomes, and were done in specific populations.

Measuring cold exposure is difficult. One used definition of cold environment at work is an ambient temperature <10°C (International Organization of Standardisation, 2008). However, contact with cold surfaces, cold liquids, draughts, humidity, and amount of clothing also affects the heat loss of an individual. The level of physical activity, and consequently heat production, might lessen the need for protection against the cold. The lack of an association for leisure time exposure might indicate that the setting of the exposure is of importance. The possibility to mitigate the negative effects of cold exposure by wearing more clothes or terminate the exposure might be higher when it is a leisure time activity. Another possibility is that cold exposure might not be a risk factor for musculoskeletal conditions, and that the association between occupational exposure and pain could be confounded by other risk factors. Workers that are exposed to cold might be more exposed to other known concurrent risk factors for pain like heavy lifting, vibration, and awkward working positions. Some of the studies try to take other occupational risk factors into account, but good measurement of all possible confounders and the statistical strength to satisfactorily adjust for these is difficult to achieve. Occupation is also considered as a marker of socioeconomic position, which again is found to be related to pain and other risk factors for pain such as level of physical activity, smoking, obesity, and poor health (Poleshuck and Green, 2008; Mills et al., 2019). Consequently, some studies could be confounded, and even studies with a comprehensive adjustment for other risk factors could be vulnerable to residual confounding.

The discussed heterogenous measurements of exposure, as well as for outcomes, impedes any meta-analysis. The estimated effect size would not be interpretable. On the other side, associations were found using many different measures of cold exposure and in many different countries. These convergent results strengthen the hypothesis that cold exposure increases the risk for musculoskeletal conditions. Another finding that strengthens the hypothesis is that some of the included studies found a dose-response relationship between cold exposure and pain (Pope et al., 1997; Raatikka et al., 2007; Sormunen et al., 2009; Burstrom et al., 2013; Stjernbrandt and Farbu, 2022). One study also found a dose-response relationship for frequency of feeling cold and chronic pain (Farbu et al., 2019). Two of these studies use a subjective measure of cold exposure, i.e., feeling cold or cooling, and the self-reported exposure in several of the other studies could be influenced by the subjective experience. A study of workers in the fishing industry could not find any simple relationship between the ambient temperature and the frequency of feeling cold and feeling cold often was associated with musculoskeletal pain (Bang et al., 2005). Thus, the subjective experience of the environment might be an important marker for the cold exposures’ effect on the body, but a poor marker of the thermal environment. On the other side, the experience of pain and the environment, as well as having pain, could very well be closely connected (Sundstrup et al., 2015; Farbu et al., 2021b). The central sensitization associated with pain could increase the risk of feeling cold or cooling, and the likelihood of reporting a cold environment. Consequently, the cross-sectional studies using self-reported exposure could be vulnerable to reverse causation.

The limited evidence indicating that cold exposure is a risk factor for pain or chronic pain identified in this review could be supported by literature that did not meet our inclusion criteria. One study noted that a colder temperature at the working place was the only notable difference between two groups with different incidence of tenosynovitis in food-processing workers (Kurppa et al., 1991), and other studies have found associations between different environmental complaints and pain (Hildebrandt et al., 2002; Magnavita et al., 2011; Sundstrup et al., 2015). A case-control study found an association between working in a cold environment and having rheumatoid arthritis (Zeng et al., 2017). Further, several experimental studies have found that cold temperature might affect neuromuscular function. For example, cold temperature caused decreased muscle power and contraction velocity (Racinais and Oksa, 2010), it increased stiffness of tendons (Alegre et al., 2016), as well as decreased nerve conductivity (Racinais and Oksa, 2010). A more direct association is found in individuals experiencing non-freezing cold injury, a neuropathic condition causing pain and sensory disturbances (Vale et al., 2017). The possible nerve swelling caused by cooling could be part of the explanation of the association between cold exposure and CTS (Ulaşli et al., 2014). These findings could indicate that cold exposure cause structural changes which increases the risk of pain. In addition, many report that their pain condition is aggravated by weather (Timmermans et al., 2014) and that cold temperature causes pain. Further, the findings that weather affects pain tolerance indicates that the climatic environment can be of importance for how and when we feel pain (Farbu et al., 2021b).

Pain is one of the most prominent symptoms of musculoskeletal conditions, and pain is an experience and a feeling, it is subjective in nature, and it seems to vary over time (Glette et al., 2020; Farbu et al., 2021b). Measuring it is therefore inherently difficult. In addition, the reporting of pain seems to be affected by the society the individual lives in (Zimmer et al., 2021). To further complicate it, the correlation between clinical findings and pain can often be poor (Dieppe and Lohmander, 2005), meaning a clinically verified diagnosis is not a good measure of an individual’s pain. Thus, the best measure of outcome might be dependent on the causal hypothesis about the association: does cold exposure increase the risk of experiencing pain or does it cause structural changes which again increases the risk of pain?

The results presented in this review are vulnerable to possible publication bias. Some of the included studies do not have a specific aim of investigating cold exposure as a risk factor. Other studies that also included cold exposure as one of many risk factors may have chosen to not report the estimates due to non-significant results. To which extent this is present is not known.

Another limitation is the use of odds ratios as an effect measure, as it has no intuitive interpretation. OR is “overestimated” if interpreted as relative risk, and this bias increases with increasing prevalence (Szklo and Nieto, 2018). The prevalence of the outcomes in the included studies ranged from under 10% to over 80%, and direct comparison of the ORs between studies is precarious. These problems also complicate the communication of the results, and the translation into the clinic.

Some of the included studies had few participants and did not have sufficient power. For example, one study calculated a prevalence ratio based on only 2 cases (Piedrahí;ta et al., 2004), and another study found an RR of 151 with 100 participants in each group (Ghani et al., 2020). This estimate is only possible if the number of events in one group is less than one or higher than 100. Nevertheless, many studies calculated many estimates. A total of 85 estimates were extracted from 16 studies, but none of the included studies adjusted for multiple testing.

The results in some of the included studies might also be affected by the healthy-worker effect (Hernán et al., 2004). Many studies use occupational exposure, and it might very well be that those vulnerable to cold exposure or developing pain already have changed their occupation. This could lead to an underestimation of the effect.

Another limitation is that many of the studies included questions on aches, stiffness, or trouble in certain regions of the body. Thus, even though we have used the term regional pain in this review, some participants might have had other symptoms than pain. Further, our list of musculoskeletal conditions was not exhaustive.

Although the evidence for cold exposure per se as a causal factor for musculoskeletal conditions is uncertain, it is in many instances relatively easy and cheap to prevent by means of better or more clothing. The association between perceived climatic environment and pain indicates that the person’s own experience of the climate might be a good indicator of cold exposure and the need for protective measures. There is a need for prospective studies, and future research should clearly state their aim and hypothesis, restrain from testing multiple hypotheses at the same time, be sufficiently powered, and try to incorporate both objective and subjective measures of the environment. Future studies could also include an assessment of important aspects of pain like intensity and duration.
