# Supplementary material for: Cold exposure and musculoskeletal conditions; A scoping review
Source: Front Physiol. 2022 Sep 1;13:934163. doi: 10.3389/fphys.2022.934163 (PMC9475294; doi:10.3389/fphys.2022.934163)
Supplement: Supplementary file 1 [file Table1.docx]

Supplementary table 1 Criteria for assessing risk of bias.

| **Outcome** | | |
| --- | --- | --- |
| **Musculoskeletal pain** | | |
| **Criterion** | **Alternative** | Score |
| Subjective symptom description | Doctor’s anamnesis (interview) Questionnaire Missing | 2 1 0 |
| Clinical investigation | Doctor’s examination Missing | 2  0 |
| Impairment of daily activities | Single item in questionnaire Missing | 1 0 |
| Impairment of work ability | Single item in questionnaire Missing | 1 0 |
| **Neuropathic pain (Carpal tunnel syndrome)** | | |
| **Criterion** | **Alternative** | **Score** |
| Subjective symptom description | Doctor’s anamnesis (interview) Questionnaire Missing | 2 1  0 |
| Clinical investigation | Doctor’s examination (Phalen, Tinel) Missing | 2  0 |
| Objective investigation | Morphological or Electrical Diagnostics Missing | 4 0 |
| Control of diagnostic methods | Method, Subject, environment  Missing | 1  0 |
| **Exposure** | | |
| **Criterion** | **Alternative** | **Score** |
| Temperature | Objective measures  Proxy by context (i.e.geographical regions, contact with items, seasons) Subjective estimates  No information | 3  2  1  0 |
| Time | Objective measures  Subjective estimates  No information | 2 1 0 |
| **Method** | | |
| **Criterion** | **Alternative** | **Score** |
| Study design | Cohort  Case-control  Cross-section | 6  4 2 |
| Selection | Response rate > 70% or falling off at follow-up less than 30%  Response rate 40-70%  Response rate < 40 / no information | 2  1 0 |
| Control of confounding factors | Yes, comperhensive  Yes, limited No / no information | 2  1 0 |
