# Supplementary material for: Cold exposure and musculoskeletal conditions; A scoping review
Source: Front Physiol. 2022 Sep 1;13:934163. doi: 10.3389/fphys.2022.934163 (PMC9475294; doi:10.3389/fphys.2022.934163)
Supplement: Supplementary file 2 [file Table2.docx]

*Supplementary table 2 Critical appraisal of individual sources of evidence. The maximum total score differs for studies with neuropathic pain and musculoskeletal pain as outcome. The studies are ordered after total score and a higher score means less risk of bias*

| **Neuropathic pain, maximum total score 24** | | | | | |
| --- | --- | --- | --- | --- | --- |
| Reference | Study design | Total score  (Max. 24) | Subscore  Outcome (Max. 9) | Subscore  Exposure (Max. 5) | Subscore  Method (Max. 10) |
| Chiang 1990 | Cross-sectional | 16 | 9 | 3 | 4 |
| Yagev 2007 | Case-control | 15 | 8 | 1 | 6 |
| Stjernbrandt (lumbar radiculopathy) 2022 | Cross-sectional | 6 | 1 | 1 | 4 |
|  |  |  |  |  |  |
| **Musculoskeletal pain, maximum total score 21** | | | | | |
|  |  | Total score  (Max. 21) | Subscore  Outcome (Max. 6) | Subscore  Exposure (Max. 5) | Subscore  Method (Max. 10) |
| Farbu 2021 | Cohort | 12 | 1 | 2 | 9 |
| Bodin (with RCS) 2012 | Cross-sectional | 11 | 4 | 2 | 5 |
| Burström 2013 | Cross-sectional | 10 | 2 | 2 | 6 |
| Dovrat 2007 | Cross-sectional | 10 | 1 | 3 | 6 |
| Milgrom 2003 | Cohort | 10 | 4 | 2 | 4 |
| Piedrahita 2004 | Cross-sectional | 9 | 2 | 3 | 4 |
| Skandfer 2015 | Cross-sectional | 9 | 1 | 2 | 6 |
| Sormunen 2009 | Cross-sectional | 9 | 2 | 1 | 6 |
| Bodin (without RCS) 2012 | Cross-sectional | 8 | 1 | 2 | 5 |
| Farbu 2019 | Cross-sectional | 8 | 1 | 2 | 5 |
| Pinar 2013 | Cross-sectional | 8 | 1 | 1 | 6 |
| Pope 1997 | Cross-sectional | 8 | 2 | 2 | 4 |
| Raatikka 2007 | Cross-sectional | 8 | 1 | 2 | 5 |
| Ghani 2020 | Cross-sectional | 8 | 1 | 5 | 2 |
| Altuntas 2020 | Cross-sectional | 7 | 1 | 4 | 2 |
| Stjernbrandt 2022 | Cross-sectional | 6 | 1 | 1 | 4 |
